# Risk Factors of Treatment-Limiting Anemia after Substitution of Zidovudine for Stavudine in HIV-Infected Adult Patients on Antiretroviral Treatment

**DOI:** 10.1371/journal.pone.0060206

**Published:** 2013-03-26

**Authors:** Thong Phe, Sopheak Thai, Chhunheng Veng, Sopheak Sok, Lutgarde Lynen, Johan van Griensven

**Affiliations:** 1 Department of Infectious Diseases, Sihanouk Hospital Center of HOPE, Phnom Penh, Cambodia; 2 Department of Clinical Sciences, Institute of Tropical Medicine (ITM), Antwerp, Belgium; Infectious Disease Service, United States of America

## Abstract

**Background:**

Anemia is the main concern among patients using a zidovudine (AZT)-based antiretroviral treatment (ART). Some studies suggested weight-adjusted AZT dosing as a way to reduce toxicity. We analyzed the risk factors associated with AZT-induced anemia in a cohort using AZT as substitution for stavudine (D4T).

**Methods:**

We retrospectively studied HIV-infected patients in a referral hospital in Phnom Penh, Cambodia between 2003 and 2011. Factors associated with AZT-related anemia requiring AZT-discontinuation within the first year after AZT initiation were analyzed using Cox regression.

**Results:**

Overall, 1180 patients, 60.5% female, were included. At AZT initiation, the median hemoglobin was 12.7 g/dL (IQR 11.7–13.9), the median weight: 51 kg (IQR 45–58) and the median time on ART prior to AZT substitution: 1.4 years (IQR 1.0–2.0). Within one year follow-up, 139 patients (11.8%) developed anemia requiring AZT discontinuation. Overall, there was no independent association of body weight with AZT discontinuation. AZT discontinuation was associated with lower hemoglobin level when starting AZT; older age and taking D4T-based ART less than one year prior to AZT. In exploratory analysis, a linear increase in risk of grade 2–4 anemia with lower body weight was seen if starting AZT substitution within less than one year of D4T-based ART.

**Conclusion:**

Our findings argue against the need of weight-based dosing of AZT to reduce anemia among patients using AZT as substitution for D4T. Whether this also applies to ART-naïve individuals remains to be assessed. Future studies with AZT dose reduction should assess efficacy and overall tolerance of AZT.

## Introduction

Over the last few years, an impressive scale-up of antiretroviral therapy (ART) has been seen in low and middle income countries (LMIC), with 6,650,000 patients on treatment in 2010 [1]. Most of the patients in these countries currently use stavudine (D4T)-containing regimens, followed by zidovudine (AZT)-based treatment [1]. One of the key clinical and operational challenges is the management of treatment-related drug toxicity. Whereas mitochondrial toxicity is the major concern with D4T, AZT use is often complicated by the occurrence of – sometimes severe – anemia [2]. Recent World Health Organization (WHO) guidelines have recommended to phase-out the use of D4T, in favor of tenofovir or AZT [3]. Consequently, millions of HIV-infected individuals on ART for prolonged time periods will replace D4T with AZT in the near future. However, studies on the incidence and determinants of anemia in LMIC in such patients are currently scarce. A number of key questions remain to be addressed.

First, there is some evidence that the risk of anemia is particularly high in patients with low body weight. In Peru, discontinuation rate of AZT-containing regimen due to toxicity in the first 120 days increased dramatically with lower baseline weight (< 60 kg) among antiretroviral-naïve patients starting ART [4]. The authors suggested that a weight-based approach for AZT dosing should be considered to reduce the occurrence of anemia. Such findings could be particularly relevant for regions like South-East Asia, where most HIV-infected patients have a body weight clearly below 60 kg. A report from a small study in Thailand demonstrated a relationship between lower body weight and lower AZT clearance, associated with more frequent side effects (gastrointestinal intolerance and anemia) [5] and current Thai guidelines recommend a dose ranging from 200 to 300 mg twice a day [6]. AZT has been found to exhibit cytotoxicity to the erythroid precursor cells in the bone marrow *in vitro* in a dose-dependent manner. This toxicity could possibly be more pronounced in individuals with a low body weight, due to higher AZT levels [7]. However, these findings are not yet generally accepted, and the current WHO guidelines still recommend the standard dose of AZT 300 mg twice a day for adult patients [3].

Another controversy relates to the effect of prior ART use before AZT initiation. Some studies have suggested that prior exposure to ART before starting AZT is protective against AZT-induced anemia [8]–[10] and that longer duration of ART use prior to starting AZT is associated with a reduced risk of anemia [11]. Possibly, this toxicity could be exacerbated by ongoing HIV-1 infection or immune activation early after starting ART [12], [13]. However, the reported association was not confirmed in other studies [14].

Despite the recent WHO recommendation, some poor countries continue to use D4T-based regimens as the preferential first line treatment due to its good short-term tolerance, the availability of a fixed-dose combination and especially the low cost compared to other regimens. In line with the Cambodian national guideline, [15], [16] D4T is still used within the first line regimen in Sihanouk Hospital Center of HOPE (SHCH), a tertiary hospital in the capital. However, by seven years of follow-up, D4T was discontinued in around 48% of patients starting ART with D4T-based regimen due to the D4T-intolerance [17] and AZT was usually used as alternative. Based on carefully collected program data over a period of seven years, we report the incidence and risk factors of AZT-induced anemia within one year after substituting AZT for D4T in adult patients on ART in Cambodia. The main purpose of this study was to determine how the risk of anemia after AZT initiation varies across patient characteristics like body weight and duration of prior ART use.

## Methods

### Study design and study population

This was a retrospective study using data routinely collected at each consultation at SHCH between March 2003 and August 2011. The SHCH is a tertiary hospital run by a non-governmental organization, situated in the capital, Phnom Penh, Cambodia. The hospital provides free care to poor patients, including treatment of opportunistic infections and ART for HIV-infected adult patients. All consecutive ART-naive patients starting ART with a D4T-based regimen and substituting AZT for D4T due to D4T-intolerance between March 2003 and July 15, 2011 with follow-up till August 15, 2011 were included. Individuals with ART exposure prior to initiation of D4T-based ART, and patients missing baseline hemoglobin and without at least one follow-up hemoglobin result were excluded.

### HIV treatment and monitoring

In line with WHO and national guidelines, [15], [18] the following ART eligibility criteria were used: 1) CD4 cells count≤200 cells/ µL; 2) WHO clinical stage 3 with CD4 cells count≤350 cells/ µL or 3) WHO clinical stage 4 irrespective of CD4 cells count. After guideline revision, ART was indicated as well for WHO clinical stage 3, irrespective of CD4 cells count, and the CD4 threshold was increased to 350 cells/ µL [3], [16]. A fixed-dose combination of lamivudine plus D4T plus nevirapine was generally initiated as preferential first line regimen. Alternative drugs (AZT, tenofovir, abacavir, efavirenz or protease inhibitors) could be used in case of contraindication or intolerance to any of these medications. Nevirapine was replaced with efavirenz in case of patients on rifampicin-containing anti-tuberculosis treatment. AZT (300 mg twice a day) was preferentially used in case of D4T-intolerance. Cotrimoxazole prophylactic treatment was indicated for all patients with WHO clinical stage 2–4 or all those with a CD4 count<200 cells/ µL. All patients with WHO clinical stage 4 diseases or a CD4 count<100 cells/ µL were started on fluconazole primary prophylaxis. Fluconazole and cotrimoxazole prophylaxis were discontinued in patients on ART when CD4 increased to more than 100 cells/ µL and 200 cells/ µL respectively.

After substitution with AZT, clinical and laboratory monitoring was done at regular intervals to detect the development of anemia or other related side-effects. Hemoglobin measurement was performed prior to AZT initiation, monthly for the first 3 months, then at 6 months and repeated every six months after that, or on clinical indication. AZT initiation was contra-indicated for patients with hemoglobin levels less than 8 g/dL and it was discontinued in case hemoglobin dropped below 6.5 g/dL or decreased more than 25% from the peak value, after ruling out other potential causes of anemia. The WHO's criteria of grading the severity of laboratory toxicity were used to define the grade of anemia [3]; grade 1: hemoglobin (Hb) 8.0 to<9.5 g/dL; grade 2: Hb 7 to<8.0 g/dL; grade 3: Hb 6.5 to<7 g/dL and grade 4: Hb<6.5 g/dL.

### Data collection and statistical analysis

All available clinical and laboratory data were prospectively recorded using standardized data collection tools and entered prospectively in a local electronic database. Quality control of the entered data was done at regular intervals using standard of procedure for quality monitoring [19].

The primary outcome was time to AZT discontinuation due to anemia (treatment-limiting or ‘severe’ anemia) within the first year after AZT initiation. Follow-up time was censored at the date of AZT discontinuation, death, last visit within the first year after AZT initiation and August 15, 2011 for the remainder. Cumulative incidence of AZT-related anemia within the first year after AZT initiation was estimated using Kaplan-Meier methods. Body weight at the time of AZT initiation was taken as main exposure, categorized into clinically meaningful categories(>60 kg, 50–60 kg, 40–50 kg and<40 kg). We constructed a Cox proportional hazard models to study the association between body weight at the time of AZT initiation and risk of AZT-related anemia within the first year of AZT use, adjusted for confounding factors. The following factors were considered *a priori* for inclusion: hemoglobin levels and CD4 cell count at time of AZT initiation, time on ART prior to AZT initiation. A number of additional factors were considered as potential confounders for inclusion in the model: use of cotrimoxazole and fluconazole concurrent with AZT initiation, age, sex, baseline WHO clinical stage.

Starting from the full model including all co-variates, a backward selection process was performed by observing the effect on the outcome of removing every individual predictor (besides the main exposure and the *a priori* identified confounders) one by one, starting with the variable with the weakest association with the outcome. Subsequently, all co-variates were added again in the model in a forward selection process to observe whether joint effects of co-variates existed. Co-variates were retained in the model if their removal/inclusion induced a change of>10% in the measure of effect of the main exposure or they were significantly associated with the outcome in adjusted analysis. Interactions were explored guided by the bi-variate analysis and current knowledge.

Since the proportional hazard assumption - tested graphically and formally using Schoenfeld residuals - was violated for the variable ‘CD4 levels at AZT initiation’, the model was stratified across CD4 count strata. In sensitivity analysis, an alternative modeling strategy was explored, including an interaction term between CD4 count and time on AZT. A number of additional exploratory and sensitivity analyses were conducted: 1) grade 2–4 anemia was taken as outcome (hemoglobin<8 g/dL): 2) main exposure was included as a binary variable (gender-specific cut-off at the median) or categorized into quartiles; 3) BMI at the time of AZT initiation was included as main exposure or confounding factor. BMI was analyzed as 1) categorized as>25 kg/m^2^, 18.5–25 kg/m^2^ and<18.5 kg/m^2^; 2) quartiles. In exploratory analysis, the association of body weight and anemia was stratified according to time on ART prior to AZT initiation. All analysis was done using STATA software version 11.0 (Stata Corporation, College Station, TX, USA). All statistical tests were two-sided, statistical significance was defined as *p*<0.05.

#### Ethical issues

For purposes of program monitoring and evaluation, and research activities, clinical and laboratory data have been routinely recorded since the HIV care program was initiated in 2003. Patients were requested to give written informed consent to store and use the data. There was no linkage of these data with other sources which could identify the individual patient. The data collection and informed consent procedure were approved by the Institutional Review Board of Institute of Tropical Medicine, Antwerp and the Institutional Review Board SHCH. No patient identifiers were included in the dataset used for this analysis.

## Results

Between March 2003 and July 2011, 2856 ART-naive adults initiated D4T-based first line ART. Of these, 1290 (45.2%) were changed to AZT due to D4T-toxicity. With 110 (8.6%) excluded due to missing hemoglobin results, either at baseline or during follow-up while on AZT, 1180 patients were included in the analysis. Of these included patients, 60.5% were female and the median age was 35 years old (interquartile range [IQR] 30–41), see Table 1. The median time elapsed between starting d4T-based regimen and AZT substitution was 1.4 years (IQR 1.0–2.0). The median weight at AZT initiation was 51 kg (IQR 45–58) and only 19.1% (227 patients) had a body weight equal or more than 60 kg. At AZT initiation the median hemoglobin was 12.7vg/dL (IQR 11.7–13.9), and 24 patients (2%) had already anemia grade 1 (hemoglobin of 8–9.5 g/dL). One year after AZT initiation, 1142 patients (96.8%) were retained on ART in the program. Of the 38 patients (3.2%) that were non-retained, 18 (1.5%) had died, 16 (1.4%) were lost to follow-up and 4 (0.3%) had been transferred out to other healthcare facilities. Within the first year of starting ART, a total of 5500 hemoglobin measurements were performed for the 1180 individuals while taking AZT, with a median of 6 (IQR 4–6) samples per patient.

**Table 1:** Characteristic of adult patients on antiretroviral treatment substituting AZT for D4T (N = 1180).

At the time of ART initiation (D4T-based)	
Age (years) – median (IQR)	35 (30–41)
Gender - n (%)	
Male	466 (39.5)
Female	714 (60.5)
WHO clinical stage - n (%)	
Stage 1–2	214 (18.1)
Stage 3–4	966 (81.9)
At the time of AZT substitution	
CD4 count, (cells/ µL) - median (IQR)	288 (186–413)
On cotrimoxazole prophylaxis - n (%)	561 (47.5)
On fluconazole prophylaxis - n (%)	262 (22.2)
Body weight (kg) - median (IQR)	51 (45–58)
Hemoglobin level (g/dL) - median (IQR)	12.7 (11.7–13.9)
Time on D4T-based ART (years) - median (IQR)	1.4 (1.0–2.0)

Status at the time of censoring (up to 1 year after substitution with AZT)	
Retained in care	1142 (96.8%)
Dead	18 (1.5%)
Lost to follow-up	16 (1.4%)
Transferred out	4 (0.3%)

IQR: interquartile range, WHO: World Health Organization, ART: antiretroviral therapy, AZT: zidovudine, D4T: stavudine

**Figure 1:**
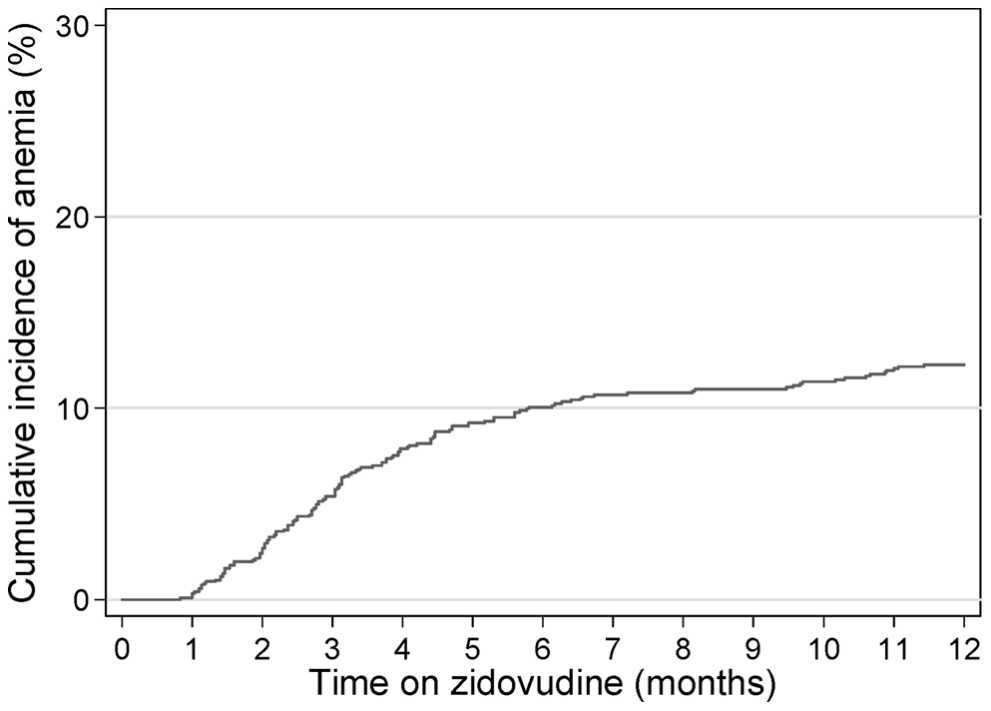
Cumulative incidence of AZT-related anemia requiring AZT-discontinuation over 1 year of AZT use.

Overall, one hundred and thirty-nine (11.8%) who were substituted with AZT experienced anemia requiring AZT-discontinuation, after a median time of 94 days (IQR 63–155). The estimated probability of AZT discontinuation was 5.3%, 10.1%, and 12.5% at 3, 6 and 12 months respectively after AZT initiation (Figure 1), with an incidence rate of 13.8 per hundred person-years. Among those who discontinued AZT, the median hemoglobin was 7.6 g/dL (IQR 5.6–8.8); and 39.6% (55/139) had anemia grade 3 or 4. At the time of censoring, 76 out of 1180 patients (6.4%) had anemia grade 1, and 88 patients (7.1%) had anemia grade 2 or more (Table 2). There were no reported deaths related to AZT-induced anemia, but about one third of those 139 anemic patients needed blood transfusion.

**Table 2:** Hemoglobin change over the time of patients on AZT.

Anemia grade	At baseline AZT	At the time of censoring; N = 1180;	At the time of AZT discontinuation; N = 139;
	N = 1180;	n (%)	n (%)
	n (%)		
0	1156 (97.8)	1019 (86.4)	13 (9.4)*
1	24 (2.2)	76 (6.4)	44 (31.6)
2	0 (0)	30 (2.5)	27 (19.4)
3	0 (0)	8 (0.7)	8 (5.8)
4	0 (0)	47 (4.0)	47 (33.8)

AZT: zidovudine

* No anemia: these patients discontinued AZT due to a drop in hemoglobin of>25%

In univariate analysis, body weight at AZT initiation and gender were not associated with anemia requiring AZT discontinuation (Table 3). There was a significantly higher risk of AZT-discontinuation for individuals starting AZT with hemoglobin levels below 12 g/dL (HR 2.2; 95% CI 1.6–3.2 for Hb 10–12 g/dL and HR 7.0; 95% CI 4.0–11.1 for Hb<10 g/dL). Older age and starting AZT after using D4T less than one year were also associated with higher risk of AZT-discontinuation (HR 1.3; 95% CI 1.1–1.6 and HR 1.8; 95% CI 1.3–2.6 respectively).

**Table 3:** Risk factors associated with AZT-induced anemia requiring AZT discontinuation.

	Event/N (%)	Univariate analysis		Multivariate analysis	
		HR	*p* value	aHR	*p* value
Body weight at AZT start					
≥60 kg	23/225 (10.2)	1		1	
50–59 kg	51/457 (11.2)	1.1 (0.6–1.7)	0.814	1.0 (0.6–1.7)	0.982
40–49 kg	51/427 (11.9)	1.1 (0.7–1.9)	0.953	1.0 (0.6–1.8)	0.916
<40 kg	13/71 (18.3)	1.7 (0.8–3.3)	0.152	1.1 (0.5–2.4)	0.788
Hemoglobin at AZT start					
>12 g/dL	66/827 (8.0)	1		1	
10–12 g/dL	53/312 (17.0)	2.2 (1.6–3.2)	<0.001	2.2 (1.5–3.3)	<0.001
<10 g/dL	19/41 (46.4)	7.0 (4.0–11.1)	<0.001	6.5 (3.7–11.4)	<0.001
Age (per 10 year increase)	139/1180 (11.8)	1.3 (1.1–1.6)	0.002	1.2 (1.0–1.4)	0.042
Gender					
Male	56/466 (12.0)	1		1	
Female	82/714 (11.5)	1.0 (0.7–1.4)	0.877	0.7 (0.5–1.1)	0.155
Time on D4T					
≥1 year	91/924 (9.9)	1		1	
<1 year	47/256 (18.4)	1.8 (1.3–2.6)	0.001	1.4 (1.0–2.1)	0.057

HR: Hazard ratio, aHR: adjusted hazard ratio, AZT: zidovudine, D4T: stavudine

Additional co-variates included in univariate analysis but not retained in multivariate analysis are described in the Methods.

In multivariate analysis, risk of AZT-discontinuation remained significantly associated with lower hemoglobin at AZT initiation (adjusted HR 2.2; 95% CI 1.5–3.3 and aHR 6.5; 95% CI 3.7–11.4 for hemoglobin between 10–12 and less than 10 g/dL respectively) and older age (aHR 1.2; 95% CI 1.0–1.4). No association with body weight at the time of AZT initiation was seen. This remained true in sensitivity analysis with alternative definitions of outcome or exposure or with an alternative modeling strategy (as described in the Methods). However, a significant interaction between body weight and time on ART prior to AZT initiation was seen when grade 2–4 anemia was taken as outcome (*p*-value 0.024), further detailed in Table 4. There was no clear difference in risk of anemia by patients' body weight if the duration of D4T use prior to AZT initiation was more than one year. Conversely, a linear increase in risk of anemia with lower body weight was seen for individuals starting AZT within one year of ART initiation. The association was statistically significant when anemia of at least grade 2 was taken as outcome but not with treatment-limiting anemia.

**Table 4:** Association of body weight with anemia according to prior duration of D4T use.

	AZT start>1 year after D4T initiation		AZT start≤1 year after D4T initiation	
	aHR	P-value	aHR	P-value
Outcome: AZT discontinuation due to anemia				
Body weight				
>60 kg	1	0.522	1	0.180
50–60 kg	0.8 (0.5–1.5)		1.8 (0.6–5.4)	
40–50 kg	0.8 (0.4–1.6)		1.9 (0.6–5.6)	
<40 kg	0.7 (0.2–1.9)		2.6 (0.7–8.9)	
Outcome: Anemia grade 2 or more (hemoglobin below 8 g/dL)				
Body weight				
>60 kg	1	0.437	1	0.031
50–60 kg	0.4 (0.2–0.9)		4.4 (0.6–35.0)	
40–50 kg	0.7 (0.3–1.4)		4.4 (0.5–35.1)	
<40 kg	0.5 (0.1–2.0)		9.5 (1.1–80.7)	


aHR: adjusted hazard ratio, AZT: zidovudine, D4T: stavudine

## Discussion

AZT is increasingly used throughout the world, especially in patients with D4T-intolerance. Whereas AZT dose recommendations have been guided by studies in the West, patients in regions like South-East Asia generally have clearly lower body weight. Some data suggested that, given the lower AZT clearance, this would put individuals with low body weight at increased risk of AZT-induced anemia [4], [5]. If confirmed, AZT dosing should be individually adapted for HIV adult patients to reduce this side effect, as currently done in Thailand where AZT dosing ranging from to 200 to 300 mg twice daily is recommended [6].

In our study, we did not find an independent association of body weight with AZT-related anemia. This finding remained true in extensive sensitivity analysis, arguing against the need of weight-based dosing, at least for ART-experienced individuals with D4T-intolerance. Our observations are in contrast with those from a study in Peru [4]. Several factors could be involved, including different outcome definitions. More importantly, they only recruited ARV-naïve individuals. In light of this, our observation in exploratory analysis of an interaction between body weight and duration of prior ART use should be considered. If AZT was started relatively shortly after starting the initial ART regimen, a negative association between body weight and AZT-associated toxicity was observed in our study, similar as the study in Peru [4]. One could hypothesize that this patient group is more ‘alike’ ARV-naïve individuals. Although we acknowledge this is speculative, it would be careful to assess this possibility in future studies. Programs now scaling-up AZT use in both ARV-naïve patients as well as those on D4T-based ART would be in a good position to address this question. With regards to ART-naïve individuals, the ongoing clinical trial comparing reduced and standard dose of AZT will be of major interest [20]. We note that differences in occurrence and risk factors of NVP-toxicity have been observed between ART-naïve and ART-experienced individuals, including in Cambodia [21], [22]. In general, more studies on how toxicity associated with specific drugs varies according to previous ART use are warranted.

Data on the effect of ART use prior to AZT initiation on the risk of subsequent anemia have also been conflicting. Whereas one study in Cambodia suggested that systematic substitution to AZT after six months of D4T-containing ART could reduce the risk of anemia [9], no clear impact was seen in another study with AZT substitution at a median of 18 months after ART initiation [14]. However, none of these studies had a concurrent control group. Some other studies observed that ARV-experience was protective against the risk of AZT-induced anemia, but the effect of duration of ART use was not specified [8]–[10]. Kumarasamy N. *et a.l* [11] reported on a cohort in India whereby a systematic prophylactic substitution of AZT for D4T was applied once the hemoglobin level had reached 11 g/dL under D4T-containing ART. In univariate analysis, patients starting AZT within six months on D4T had significantly lower hemoglobin levels than those who had substituted AZT after 6–12 months on D4T [11]. Differences in outcome and study population between the different studies could have contributed to the conflicting results. Our data are suggestive of a reduced risk of anemia if AZT is initiated after at least one year of D4T-containing ART. However, the effect was small and only reached statistical significance in univariate analysis, suggesting that other factors changing over time like hemoglobin levels could mediate this effect. Moreover, our data only apply to individuals initiating AZT because of D4T-intolerance. Scaling-up of AZT will provide the opportunity to study the effect of duration of prior ART use on AZT-related anemia in more detail.

We also found older age and lower hemoglobin level to be significantly associated with an increased risk of anemia. A wide range of risk factors associated with AZT-induced anemia have been reported in literature including older age, advanced stage of HIV infection, lower CD4 lymphocyte count, lower hemoglobin level at the time of starting AZT-containing regimen, female gender, patient's BMI and concomitant use of cotrimoxazole prophylaxis [4], [8], [14], [23], [24]. Nevertheless, there has been substantial inconsistency across the different studies, which requires further clarification.

Our study has a number of limitations. First, as a retrospective study using routinely collected program data, data quality might be a concern. However, standard data collection forms were used and the data were prospectively entered into a database with regular quality monitoring. Moreover, a standardized treatment protocol was in place. Although treatment decisions for clear-cut cases could be taken by the individual physician, management of more complicated cases was discussed in team. Second, although all individuals were systematically evaluated for alternative causes of anemia, investigations available in our setting to rule out e.g. hematological conditions or nutritional deficiencies were relatively limited. We acknowledge that the reported cases of AZT-discontinuation due to anemia in this study or presumed to be related to AZT, were without proven causal link. Similar constraints have applied to most other reported studies. Moreover, viral load testing was not done routinely. However, we previously observed very low rates of virological failure in this population [25]. Also, in our cohort as in most other Asian populations, most patients' weight was below 60 kg. This could in part have contributed to the overall high rate of anemia in our cohort [4], [5]. Finally, our data only apply to the specific patient population initiating AZT because of D4T-intolerance.

In conclusion, there was no association between patients' body weight and the development of AZT-related anemia requiring AZT-discontinuation among patients using AZT as substitution for D4T due to D4T-intolerance. This argues against the need of weight-based dosing of AZT for this patient population as a way to decrease the incidence of AZT-related anemia. However, it needs to be further explored whether this is also true for ARV-naïve individuals or in case AZT substitution is done shortly after the initiation of ART. Given the global scaling-up of AZT use, additional studies from different settings and populations are needed to provide additional information on how the risk of AZT-related anemia varies according to previous ART use and body weight [3]. More generally, it remains to be assessed whether, while keeping similar efficacy, lower AZT dosing would be associated with increased tolerance and a reduced incidence of other side-effects like early intolerance (headache and nausea) and long-term mitochondrial toxicity. Nevertheless, given the overall high rate of anemia associated with AZT, even following initial treatment with D4T, our findings also argue for increased use of tenofovir in first line ART regimens.
